# Grade IV hiatal hernia in a neonate: A radiologic mimic of congenital diaphragmatic hernia

**DOI:** 10.1016/j.radcr.2025.08.066

**Published:** 2025-09-22

**Authors:** Raed Al Taher, Somaya Al Kiswani, Mohammad Al Na’seh, Haneen A. Banihani, Omar Sawafta, Ameer Awashra, Islam Rajab, Raneem Othman

**Affiliations:** a Pediatric department, Jordan university hospital, Amman, Jordan; b Radiology department, King Hussein Cancer Center, Amman, Jordan; c General Medicine, North West Anglia NHS Foundation Trust, Peterborough, UK; d Faculty of Medicine, University of Jordan, Amman, Jordan; e Department of Medicine, An Najah National University, Main Street, Nablus 12345, Palestine

**Keywords:** Congenital Hiatal Hernia, Neonatal Imaging, Diaphragmatic Anomalies

## Abstract

Congenital hiatal hernia is a rare condition in neonates, often presenting with respiratory distress and mimicking other thoracic or diaphragmatic anomalies such as congenital diaphragmatic hernia. In this report, an 8-day-old female neonate was initially suspected of having congenital diaphragmatic hernia based on imaging studies but was ultimately diagnosed with a Grade IV congenital hiatal hernia. Surgical exploration revealed significant herniation of abdominal organs into the thoracic cavity without diaphragmatic defects, confirming the diagnosis. Multiple diagnostic modalities, including barium swallow radiography and computed tomography, were pivotal in identifying the condition, emphasizing their utility in distinguishing hiatal hernias from other mediastinal pathologies. Comprehensive surgical management, including reduction of herniated contents and Nissen fundoplication, effectively addressed the hernia and its complications. The case illustrates the variability in clinical presentation and diagnostic challenges associated with hiatal hernias, highlighting the importance of tailored approaches for diagnosis and treatment.

## Introduction

Hiatus hernia (HH) is common in adults but rare in children and neonatal period. It involves the displacement of abdominal structures, other than the esophagus, into the thoracic cavity through an enlarged diaphragmatic hiatus. In adults, HH often develops as a complication of antireflux surgery due to extensive dissection of the hiatus. In pediatric cases, however, it is typically primary or congenital and may contribute to gastroesophageal reflux. Although uncommon, congenital hiatal hernia can lead to respiratory distress, mimicking other conditions, which necessitates prompt and accurate differentiation for effective management. In this case, an initial suspicion of congenital diaphragmatic hernia was ultimately identified as a hiatal hernia, underscoring the critical role of detailed imaging for precise diagnosis, management of congenital hiatal hernia can be surgical or laparoscopic, involving hernia reduction, sac excision, crural closure, and an antireflux procedure to prevent GERD though complications like gastric perforation, dysphagia, and hernia recurrence may occur [1,2].

While hiatus hernia is frequently observed in adults, it is uncommon in children and extremely rare in its congenital form. This study presents a case of an 8-day-old female neonate presented with respiratory distress and was diagnosed with a type IV congenital hiatal hernia involving herniation of abdominal contents. Surgical correction and Nissen fundoplication were performed, along with management of concurrent PUJ obstruction causing hydronephrosis. Recovery was smooth with normal development on follow-up.

## Case presentation

**Fig. 1:**
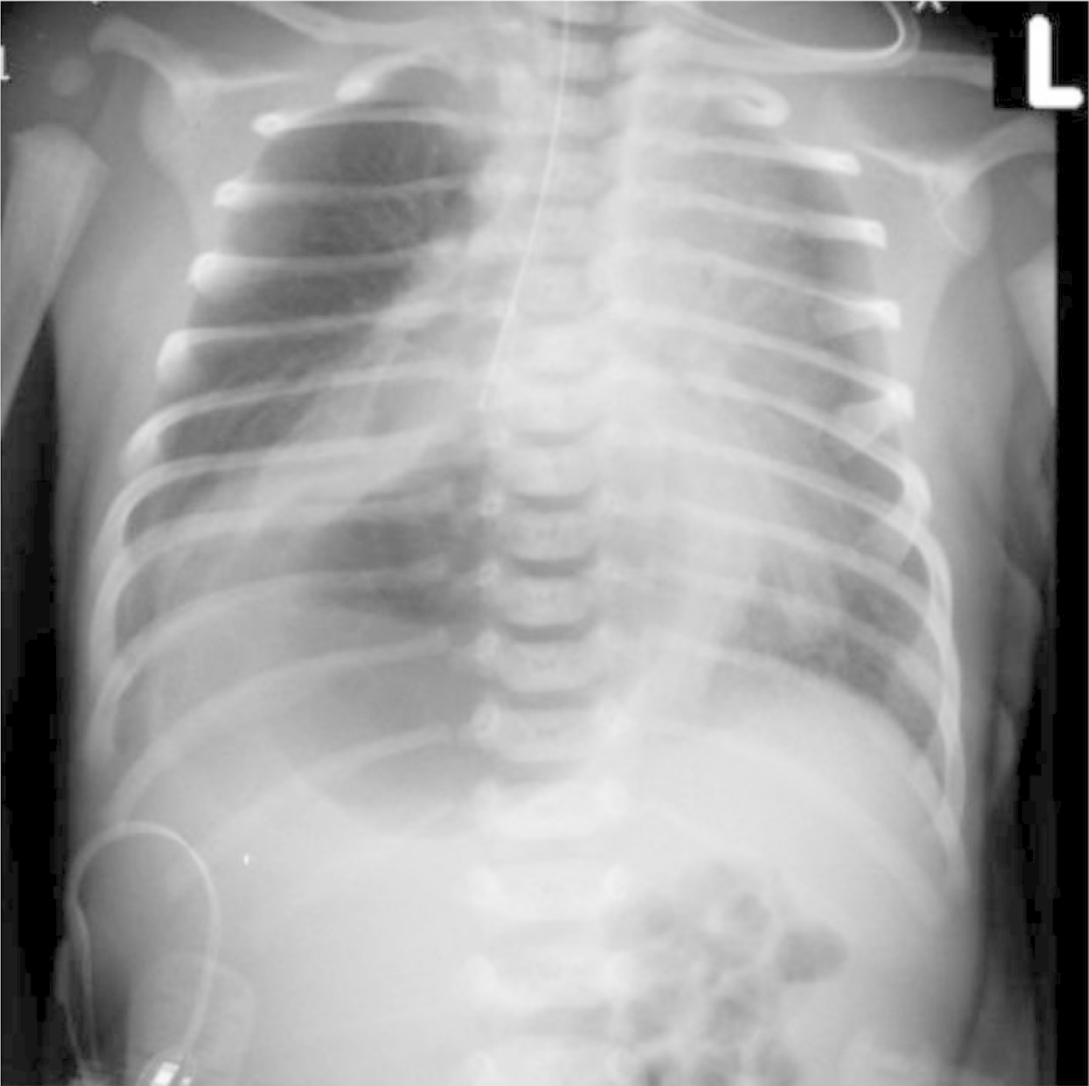
Initial chest X-ray at birth shows increased lucency of the right hemithorax, raising differential diagnoses including pulmonary sequestration and congenital lobar emphysema. A gastric bubble projecting into the thoracic cavity is evident, consistent with a congenital hiatal hernia.

A 8 days female neonate was prenatally suspected of having a congenital hernia based on imaging findings. She presented shortly after birth with significant respiratory distress, initially raising concerns for congenital pulmonary airway malformation (CPAM), congenital lobar emphysema, or a concurrent hiatal hernia. On admission to the NICU, a comprehensive physical exam was performed, including assessment of respiratory effort, skin color, muscle tone, reflexes, and abdominal distension to identify any immediate abnormalities. A preliminary chest X-ray revealed right-sided increased lucency with a visible gastric bubble projecting into the thoracic cavity, suggesting a congenital hiatal hernia ***(***Fig. 1***).***

**Fig. 2:**
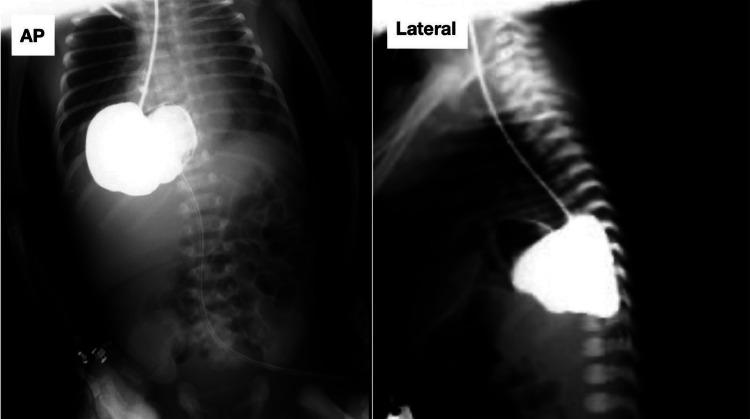
Barium study demonstrates an opacified stomach abnormally positioned within the right hemithorax through a posterolateral diaphragmatic defect, consistent with a congenital hiatal hernia. The exact herniated contents could not be fully determined at this stage, with possible involvement of the spleen or bowel loops.

**Fig. 3:**
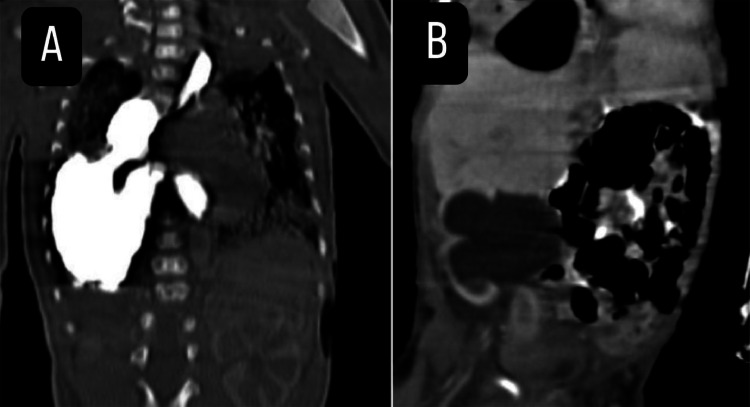
Coronal CT scan (A) demonstrates a large right-sided posterior diaphragmatic hernia containing a significant portion of the stomach and likely small bowel loops, with marked displacement of the heart and mediastinum to the left. Coronal CT scan (B) shows severe right-sided hydroureteronephrosis extending to the urinary bladder.

To further investigate, a barium study ***(***Fig. 2***)*** and comprehensive chest and abdominal CT scan with oral and intravenous contrast was performed ***(***Fig. 3***)***. The imaging findings were initially interpreted as a primary congenital diaphragmatic hernia and patchy bilateral ground-glass opacification involving both lungs and Severe right-sided hydroureteronephrosis, prompting an urgent consultation with pediatric surgery. Given the severity of the symptoms, a decision was made to stabilize the patient and proceed with exploratory laparotomy.

During surgery, a Kocher incision was extended from the left subcostal margin. Contrary to the initial diagnosis, intraoperative findings revealed a type IV hiatal hernia involving significant herniation of abdominal contents, including the left lobe of the liver, spleen, transverse colon, and gastric fundus into the thoracic cavity. Notably, no diaphragmatic defect was observed, effectively ruling out congenital diaphragmatic hernia and suggesting a congenital hiatal hernia. The surgical team performed a reduction of the herniated contents followed by a 360-degree Nissen fundoplication to prevent gastroesophageal reflux.

Postoperative recovery was smooth; the nasogastric (NG) tube was removed on the fourth day, and the patient demonstrated good tolerance to feeding, normal bowel movements, and a soft, nondistended abdomen. Predischarge evaluations focused on the renal anomaly detected during surgery. A renal ultrasound revealed severe right-sided hydronephrosis with cortical thinning and an enlarged renal pelvis measuring 28 mm in the anteroposterior dimension. The left kidney appeared normal in size and function. Subsequent cystography did not reveal any bladder abnormalities. Given the degree of hydronephrosis, a follow-up renal ultrasound was conducted 3 weeks later, which confirmed persistent findings with further dilatation of the right renal pelvis.

To assess renal function, a MAG3 renogram was performed, showing a severely hydronephrotic right kidney with preserved renal function but no effective excretory response, raising suspicion for pelvi-ureteric junction (PUJ) obstruction. Based on these findings, surgical intervention was planned. Two months later, the patient underwent a successful excision of the stenotic PUJ, reduction of the dilated renal pelvis, and placement of a size 12 JJ stent in the right ureter to ensure drainage. The JJ stent was removed 4 months postoperatively using a dormia basket without complications.

Currently, the patient is thriving with no signs of recurrent hernia or renal dysfunction. Follow-up assessments have shown normal growth and development, with parameters consistent with her age group. Ongoing monitoring is planned to ensure continued renal function and to detect any potential long-term complications associated with the hiatal hernia repair and PUJ obstruction.

## Discussion

Hiatal hernia is a condition in which the gastroesophageal junction or other abdominal viscera protrude through the esophageal hiatus of the diaphragm into the mediastinu [3,4]. It is either congenital or caused by increased intra abdominal pressure and an imbalance in the pressure gradient between the thoracic and abdominal cavities [[3], [4], [5]]. Congenital defects in children are the leading cause of paraoesophageal hernias [6], and the main risk factors for developing a hiatal hernia are increased age and obesity [3,5,7].

The table below summarizes the 4 types of hiatal hernias (Table 1), categorized based on the anatomical displacement of the gastroesophageal junction, stomach, and other abdominal organs. Each type highlights distinct features that aid in diagnosis and management [[3], [4], [5]].

**Table 1:** Overview of the types of hiatal hernias.

Type	Description	Key features
Type I	Sliding hiatal hernia. The gastroesophageal junction and the gastric cardia are above the diaphragm.	Comprises about 90% of all hernias.
Type II	Rolling or pure paraesophageal hernia. The gastroesophageal junction remains in its appropriate location, but the gastric fundus is herniated.	Gastroesophageal junction stays in place; gastric fundus is herniated.
Type III	Mixed hiatal hernia. Both the gastroesophageal junction and the gastric fundus are herniated.	Both gastroesophageal junction and gastric fundus herniated.
Type IV	The stomach and other intra-abdominal organs are herniated above the diaphragm.	Involves herniation of multiple intra-abdominal organs.

Hiatal hernias could either be asymptomatic or present with a variety of symptoms. They most commonly cause gastroesophageal reflux resulting in heartburn and regurgitation. They could also present with dysphagia and epigastric or chest pain. Additionally, nonspecific symptoms like anemia and failure to thrive can be present. Larger defects may occupy significant space in the thorax and result in respiratory symptoms like shortness of breath, asthma, and bronchitis. Furthermore, paraoesophageal hernias in pediatric patients might cause respiratory distress and emesis. Acute complications of hiatal hernias include gastric obstruction, volvulus, incarceration, and strangulation [3,4,8].

Among the differential diagnoses for a cystic mass in the posterior mediastinum are pulmonary pathologies, mediastinal pathologies, and diaphragmatic hernias (eg, Morgagni and Bochdalek hernias). Hiatal hernias could be distinguished from diaphragmatic hernias on chest x-rays as they would be located centrally on the anteroposterior view and posteriorly on the lateral view [8,9].

We have reviewed multiple pieces of literature highlighting various cases of congenital and acquired hiatal hernias in pediatric patients. The cases span diverse age groups, clinical presentations, diagnostic approaches, and management strategies, providing valuable insights into the complexity and variability of this condition. The table below summarizes key findings from these studies (see Table 2).

**Table 2:** Comparative analysis of literature on congenital and acquired hiatal hernias in infants and young children.

Study	Patients	Diagnosis	Symptoms	Type of hernia	Management	Outcomes
Embleton et al., [2]	7 newborns (3 males, 4 females), diagnosed between 2010–2016, ages 4–41 months	1 case diagnosed prenatally (thoracic cyst mass on ultrasound); 6 postnatally (AP/lateral X-rays, GI series, CT)	Cough, vomiting, failure to thrive; 1 detected incidentally	4 Type IV, 2 Type III, 1 Type I	5 laparotomy, 2 laparoscopy (1 converted to laparotomy due to gastric perforation)	Uneventful outcomes reported; details of complications for individual cases not specified
Herek et al., [1]	13-month-old girl	CT showed stomach, small intestines, and transverse colon in chest; unable to differentiate between hiatal hernia and congenital diaphragmatic hernia	Vomiting, hematemesis, respiratory distress; prior GERD treatment at 2 months	Grade IV	Hiatal repair with Thal fundoplication and posterior gastropexy	Discharged on day 6; no postoperative issues on 3-month follow-up
Bataineh et al. [10]	16-month-old boy	Misdiagnosed as congenital diaphragmatic hernia or diaphragm eventration; laparoscopy revealed herniation of gastroesophageal junction and transverse colon	Symptoms not detailed	Grade IV	Reduction of herniated contents, GEJ repositioned below diaphragm using cotton sling, hernia sac excised	Discharged 1 day postop; uneventful follow-up at 1 month and 1 year
Imamoglu et al.[12]	5 infants (3 females, 2 males), ages 4–11 months	Diagnosed with right-sided paraesophageal hernias (X-rays and clinical assessment)	Obstruction in 3 cases (2 at pylorus, 1 at GEJ)	4 Mixed (Type III), 1 Pure Rolling (Type III)	All treated with laparotomy; viscera reduced, hernia sac excised	4 uneventful recoveries, discharged after ∼8 days; 1 death due to lung damage from repeated chest infections

Multiple modalities can be used for the diagnosis of a hiatal hernia. Barium swallow radiography, computed tomography (CT), endoscopy, and manometry are the most used diagnostic investigations. Barium swallow radiography allows for visualization of the hernia by showing contrast-filled stomach parts in the mediastinum, and it also shows the size of the hernia and the location of the gastroesophageal junction. Furthermore, it can detect complications like gastric volvulus and esophageal dysfunction or stenosis. However, the main disadvantage of barium swallow radiography is that it exposes patients to radiation. Computed tomography, while not routinely recommended, may give further information about the size and location of the hernia and help distinguish it from pulmonary or mediastinal pathologies . However, while it may detect complications like volvulus, it is generally not deemed superior to barium swallow radiography; the recommendation is that only 1 of these 2 modalities should be used to prevent unnecessary radiation exposure [3,7,8].

Endoscopy can diagnose hiatal hernias by measuring a greater than 2 cm gap between the squamocolumnar junction and the esophagogastric junction. It can also detect esophageal complications like esophagitis, Cameron’s ulcers, and Barrett’s esophagus. However, it may give inconsistent measurements, especially with the esophagogastric junction’s natural mobility during swallowing and breathing, and the size of the hernia may be exaggerated due to excess air insufflation during endoscopy [3,5,7].

Lastly, esophageal manometry could also diagnose hiatal hernia and rule out esophageal motility disorders like achalasia. However, it requires skilled professionals to place the manometry catheter and analyze its readings. In pediatric patients, anteroposterior and lateral chest x-ray images are appropriate for initial diagnostic evaluation, primarily due to more respiratory complications in this age group. In contrast, chest x-rays may not be the most sensitive investigation to diagnose a hiatal hernia, as they could mimic the appearance of a cystic mass in the posterior mediastinum. In addition, the hernia may also present with an abnormally located intrathoracic nasogastric tube [3,5,11].

The management of congenital hiatal hernia can be approached either surgically or laparoscopically, depending on the specific needs and condition of the patient. When opting for a surgical approach, 4 critical steps should be considered to minimize the risk of morbidity and the likelihood of requiring additional surgeries: reduction of the hernia's content, excision of the hernial sac, crural closure, and an antireflux procedure. These steps are essential for effectively treating the hernia and its associated complications. Notably, failure to perform the antireflux procedure has been linked to a 4-fold increase in the risk of developing gastroesophageal reflux disease (GERD), necessitating subsequent antireflux surgery. Several techniques are employed for crural closure and mesh reinforcement, including hiatal re-approximation using pledged, nonabsorbable sutures placed both anterior and posterior to the esophagus [3,7,8].

In cases where a laparoscopic procedure is preferred, initial decompression of gastric contents is performed using a nasogastric tube. The patient is positioned in reverse Trendelenburg, and a 5mm trocar is introduced through the umbilicus to facilitate the insertion of a 45-degree laparoscope along with 4 additional instruments, including a flexible liver retractor. Upon entry, the surgeon evaluates the degree of the hernia and its contents, which are then reduced into the abdomen. The hernial sac is excised to ensure adequate visualization of the crura. A retroesophageal window is created to facilitate the passage of the fundus for fundoplication while ensuring the vagus nerve remains protected. During fundoplication, a fold of the gastric fundus is mobilized from the left to the right side and positioned posteriorly to the esophagus. A bougie is passed through the hiatus to guide the creation of a 2cm fundoplication, during which the anterior and posterior folds of the fundus are sutured together around the esophagus. After completing the procedure, the bougie is carefully removed. This approach ensures effective anatomical correction and minimizes postoperative complications [3].

Postoperative management begins with the initiation of feeding 6 to 12 hours after surgery, with gradual advancement to full feeding within 24 hours. The mean hospital stay is approximately 1.1 days, and most patients are discharged by postoperative day 2 or 3, ensuring a swift recovery process for the majority of cases. On the other hand the postoperative complications, while relatively uncommon, can occur and should be closely monitored. The most frequent complication is gastric perforation, reported in 3–7% of cases. Other documented complications include dysphagia, pneumothorax, bowel obstruction, intussusception, wound infection, gastroparesis, and recurrence of the hiatal hernia. These potential risks underscore the importance of meticulous surgical technique and vigilant postoperative care [3,5,7].

## Conclusion

In conclusion, congenital hiatal hernia, although rare in neonates, can present with nonspecific symptoms that mimic other thoracic or diaphragmatic anomalies, posing diagnostic challenges. This case emphasizes the importance of utilizing a combination of imaging modalities to achieve accurate differentiation from conditions such as congenital diaphragmatic hernia. Surgical intervention remains the cornerstone of management, with reduction of herniated contents and antireflux procedures, such as Nissen fundoplication, being critical to prevent complications. A multidisciplinary approach, involving careful diagnostic evaluation and individualized treatment strategies, is essential to optimize outcomes and ensure long-term health in affected patients. Regular follow-up is recommended to monitor for potential recurrence or complications.

## Patient consent

Written informed consent was obtained from the patients for their anonymized information to be published in this article.
